# Configurational Entropy and Phase Stability in Lead-Free Mixed-Halide CsSn(Br_*x*_I_1–*x*_)_3_

**DOI:** 10.1021/acs.jpclett.6c00224

**Published:** 2026-03-20

**Authors:** Xing Liu, Bowen Wang, Jiacheng Gong, Xuan Chen, Yongqing Cai

**Affiliations:** Joint Key Laboratory of the Ministry of Education, Institute of Applied Physics and Materials Engineering, University of Macau, Avenida da Universidade, Taipa, Macau 999078, China

## Abstract

Metal halide perovskites have high compositional tunability, but halide mixing is often accompanied by phase segregation and instability in lead-free Sn-based systems. Here, we investigate the thermodynamics of Br/I alloying in CsSn­(Br_*x*_I_1–*x*_)_3_ by combining density functional theory calculations with partition functions over all symmetry-inequivalent configurations of the cubic, tetragonal, and orthorhombic phases. We find that the orthorhombic phase exhibits the lowest mixing free-energy curve and is closest to the thermodynamic miscibility boundary, whereas the cubic phase remains the least favorable for Br/I mixing. At 300 K, the free-energy difference Δ*F*_cub‑orth_ = *F*_cub_ – *F*_orth_ is positive over the entire composition range ((1.27–3.42)*k*_B_ *T*), indicating a robust thermodynamic preference for the orthorhombic phase. The enhanced stability of the low-symmetry phase originates from more effective local structural relaxation. Our results further reveal a link between local octahedral distortions and thermodynamic stability, providing theoretical guidance for the compositional design of lead-free Sn-based mixed-halide perovskites.

## Graphical abstract

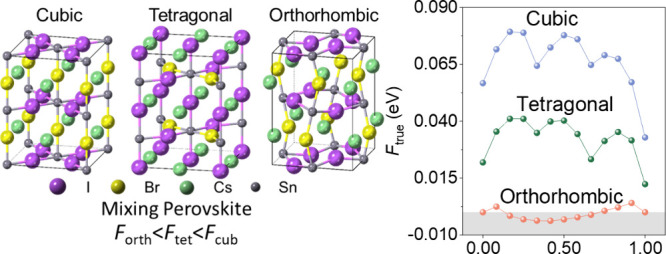

Metal halide perovskites (HPs), as a highly versatile class of emerging functional materials, have demonstrated great potential for optoelectronic devices,^1–4^ most notably in perovskite solar cells with power conversion efficiencies exceeding 27% ^5–7^ as well as in light-emitting diodes^8,9^ and detectors.^10,11^ The ABX_3_ perovskite structure features flexible crystal chemistry and solution processability.^12^ In lead-based HPs, compositional engineering has been widely employed to enhance material stability and photovoltaic performance.^13,14^ By tuning the A-site, B-site, and X-site components, the lattice constant, band gap, and carrier transport properties can be widely tuned.^15–17^ Sabino et al. investigated alloys at the A-, B-, and X-sites in HPs, demonstrating that compositional engineering at the sublattices provides an effective approach to band gap tuning.^18^ Experimentally, Shen et al. demonstrated that B-site doping with smaller Al^3+^ cations can effectively suppress lattice distortion and enhance the stability of high-entropy perovskites.^19^ However, although compositional engineering has improved device performance, it also introduces instability, particularly phase segregation due to thermodynamic and kinetic processes.^20–22^

Most high-efficiency HPs still rely on toxic lead as the B-site cation, which can generate water-soluble lead compounds, leading to environmental contamination and safety risks.^23^ Therefore, replacing lead with low toxicity elements like tin has become a focus in perovskite research.^24,25^ Sn-based HPs have demonstrated high carrier mobility, large absorption coefficients, and low exciton binding energies.^26–28^ CsSnI_3_ and CsSn­(Br_*x*_I_1–*x*_)_3_ show excellent optoelectronic properties and band gap tunability in experiments, making them important systems for investigating alloying behavior.^29–33^ However, challenges such as Sn^2+^ oxidation and phase segregation in mixed-halide perovskites (MHPs) suppress the stability and optoelectronic properties.^34^ Despite experimental progress, studies on the mixed-halide Sn perovskite phase behavior and thermodynamic stability remain limited.^35–38^

Density functional theory is widely used to study perovskite alloys and other materials,^39–45^ but the large number of configurations in MHPs makes accurate first-principles calculations challenging.^46–48^ For example, Dalpian et al. used models of cubic [FA, Cs]­[Pb, Sn]­I_3_ to evaluate composition-dependent formation energies and band gap bowing.^49^ Existing studies typically approximate the free energy of MHPs by combining a minimum mixing enthalpy (*U*_min_) with an ideal-solution mixing entropy (*S*_ideal_), which often underestimates the true free energy and oversimplifies the configurational space. Jiang et al. applied this method to the γ phase of ASn­(Br_*x*_I_1–*x*_)_3_ (A = Cs, Rb), where they approximated the mixing internal energy by considering only two representative Br/I configurations at each composition.^50^ However, single configuration energies combined with *S*_ideal_ are insufficient to capture the true thermodynamic behavior of the MHPs. To more accurately evaluate the relative stability of different phases, it is necessary to construct the partition function and compute the thermodynamic free energy (*F*_true_) and configurational entropy (*S*_confi_).^51–53^ Park et al. evaluated the formation energy, entropy, and free energy associated with X-site alloying in the orthorhombic γ phase of CsSn­(Br_*x*_I_1–*x*_)_3_ and constructed a γ–*δ* polymorph phase diagram based on free energy.^54^ Pan et al. enumerated all nonequivalent configurations of CsPb­(I_1–*x*_Br_*x*_)_3_ and CsPb­(Br_1–*x*_Cl_*x*_)_3_ to construct the partition function and associated thermodynamic state functions, thereby evaluating the temperature- and system-dependent *S*_confi_.^55^ Understanding phase stability in Sn-based MHPs remains limited. In particular, the free-energy landscape of chemical mixing is critical for composition engineering of lead-free perovskites to enhance stability and performance.

In this work, CsSn­(Br_*x*_I_1–*x*_)_3_ is employed as a model Sn-based MHP to explore the thermodynamics of Br/I alloying. All symmetry-inequivalent configurations of cubic, tetragonal, and orthorhombic phases are enumerated at each composition, and their mixing enthalpy (Δ*H*), band gaps (*E*_*g*_), and structural parameters are evaluated. An analysis of the configurational energy distributions reveals that Δ*H* is primarily governed by local structural distortions and compositional fluctuations, while global geometric parameters are unable to distinguish the energetic differences. Based on all nonequivalent configurations, the partition function is constructed to obtain the thermodynamic free energy, and the performance of three thermodynamic models in describing the mixing behavior and miscibility is assessed. Finally, by comparison of simple averaging and thermal averaging volumes and band gaps, the necessity of partition-function-based treatment is demonstrated.

For each composition (*x* = 0–1 in CsSn­(Br_*x*_I_1–*x*_)_3_), all nonequivalent structures were fully relaxed as described in the Supporting Information. CsSnI_3_ structures of the cubic, tetragonal, and orthorhombic phases are shown in Figure 1a. Figure 1b–d shows the Δ*H* for the cubic, tetragonal, and orthorhombic phases, respectively, illustrating how the Δ*H* evolves with the addition of bromide substitution over iodine.

**Figure 1:**
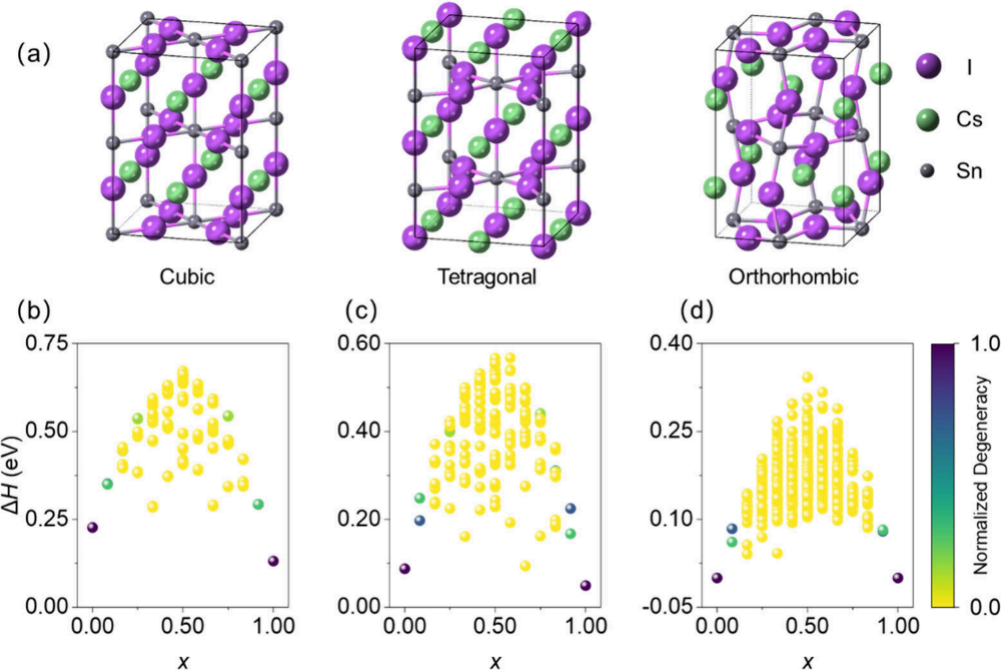
(a) CsSnI_3_ structures of cubic, tetragonal, and orthorhombic phases. (b-d) Composition dependent Δ*H* of CsSn­(Br_*x*_I_1–*x*_)_3_ at 0 K for the cubic (b), tetragonal (c), and orthorhombic (d) phases. Each sphere corresponds to a symmetry-inequivalent configuration, with the color representing normalized degeneracy.

The three phases exhibit similar energetic trends, and Δ*H* exhibits a nearly symmetric trend as a function of *x*. Δ*H* is minimized at the I-rich and Br-rich end members (*x* = 0 and 1) and increases at intermediate compositions. The positive Δ*H* for Br/I substitution indicates thermodynamic instability at 0 K. Therefore, stabilizing intermediate compositions requires compensation by entropy in the free energy at high temperature.

For the cubic phase (Figure 1), the composition with the lowest Δ*H* (131.4 meV) is Cs_4_Sn_4_Br_12_ (*x* = 1), whereas the structure with the highest Δ*H* (671.6 meV) is Cs_4_Sn_4_I_6_Br_6_ (*x* = 6/12), spanning an energy spread of 540.2 meV. In the tetragonal phase, Cs_4_Sn_4_Br_12_ (*x* = 1) has the lowest Δ*H* (49.3 meV) and Cs_4_Sn_4_I_5_Br_7_ (*x* = 7/12) has the highest Δ*H* (567.4 meV), corresponding to an energy spread of 518.3 meV. For the orthorhombic phase, the lowest-energy structure is still Cs_4_Sn_4_Br_12_ (*x* = 1) with a Δ*H* of zero and the highest-energy structure is Cs_4_Sn_4_Br_6_I_6_ (*x* = 6/12) with a Δ*H* of 342.3 meV, resulting in an energy spread of 342.3 meV. It indicates that the orthorhombic phase exhibits the highest alloy miscibility, originating from its lower lattice symmetry. As the lattice symmetry decreases from cubic to tetragonal to orthorhombic, the distribution of Δ*H* becomes narrow and octahedral tilting emerges, indicating that it is less sensitive to the specific Br/I arrangements. The low-symmetry structure can more effectively relax the local strain induced by different Br/I arrangements, leading to a lower configurational energy landscape.

As shown in Figure 2, the following analysis focused on the orthorhombic phase, while the same analysis for cubic and tetragonal phases is provided in Figures S1–4. In Figure 2a, for all orthorhombic configurations, larger *σ*(*k*), reflecting stronger local Br/I compositional fluctuations, correlates with higher Δ*H* while the lowest-energy configurations at each composition are concentrated within the low *σ*(*k*) region of 0–0.58. It indicates that strong spatial compositional inhomogeneity leads to an increasing Δ*H* and reduced alloy stability. We also employ the short-range order parameter (SRO) to analyze the local distribution of bromide and iodine. Figure 2b and Figure S5a show the relationship between Δ*H* and ⟨*Q*_Br|I_⟩ and ⟨*Q*_I|I_⟩ in the orthorhombic phase, which indicates that low-energy configurations are primarily concentrated in the region with ⟨*Q*_Br|I_⟩ > 1 and ⟨*Q*_I|I_⟩ < 1, indicating that the local Br fraction around I is higher than the overall average whereas I–I self-clustering is strongly suppressed. It indicated that the system energetically prefers heterogeneous clustering between Br and I and avoids the homogeneous clustering.

**Figure 2:**
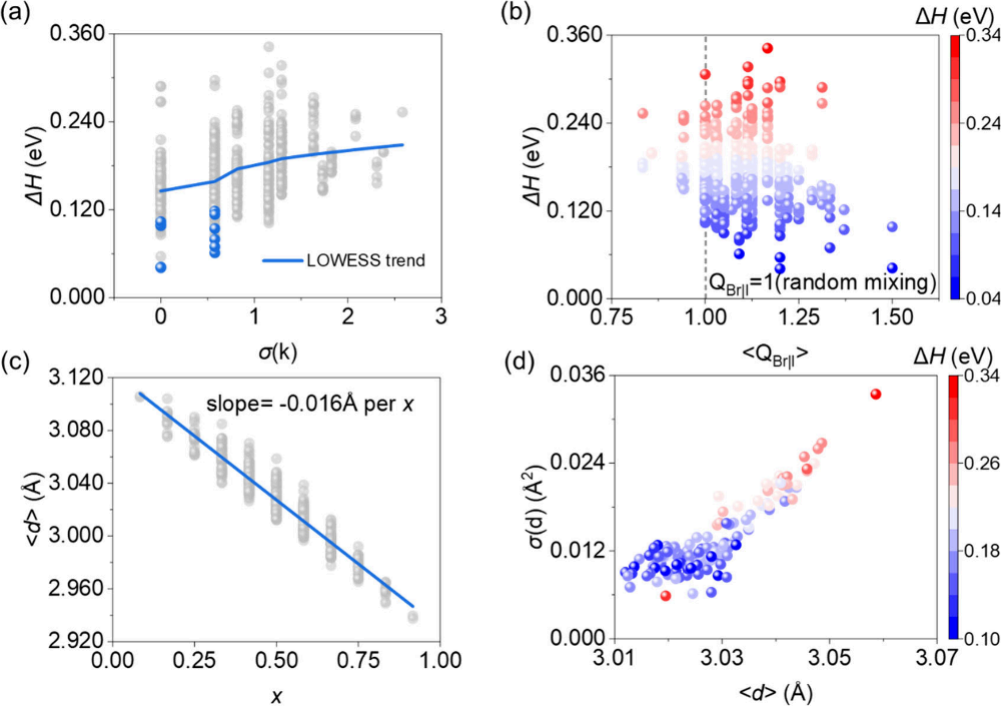
Correlations between local composition, SRO, global geometry parameters, and Δ*H* for orthorhombic CsSn­(Br_*x*_I_1–*x*_)_3_. (a) Δ*H* versus *σ*(*k*); gray and blue spheres denote all nonequivalent configurations and the lowest-energy configurations at each composition, respectively. The blue line shows a LOWESS smoothing (fraction = 0.6). (b) ⟨*Q*_Br|I_⟩ versus Δ*H*. (c) ⟨*d*⟩ versus *x*; gray spheres denote all nonequivalent configurations, while the blue line indicates a linear fit to ⟨*d*⟩. (d) Distribution of Δ*H* for Cs_4_Sn_4_I_6_Br_6_ (*x* = 6/12) configurations in the (⟨*d*⟩, *σ*(*d*)) space.

Figure 2c shows that the average Sn–X bond length ⟨*d*⟩ decreases nearly linearly with increasing *x*. At a fixed composition, the variation of ⟨*d*⟩ among different configurations is smaller than that across different values of *x*, suggesting a limited influence of specific Br/I arrangements on global structural parameters. Figure S5b shows a broad distribution of tolerance factors at each fixed composition, indicating that global geometric parameters are insufficient to describe the energetic differences among configurations, which are mainly reflected by local structure distortions and Δ*H*.

Figure 2d shows the Δ*H* distribution of all orthorhombic Cs_4_Sn_4_I_6_Br_6_ (*x* = 6/12) configurations in (⟨*d*⟩, *σ*_*d*_) space, where *σ*_*d*_ represents the standard deviation of the Sn–X bond lengths. The lowest-energy configurations cluster at shorter ⟨*d*⟩ and smaller *σ*_*d*_, indicating that energetically favorable configurations are associated with relatively uniform distributions. In contrast, the cubic phase (Figure S1d) exhibits a different distribution due to its strictly constrained degrees of freedom while the tetragonal phase shows a more scattered energy distribution, reflecting an intermediate ability to relax local strain. Figure 2a–d demonstrates that the increasing Δ*H* is mainly associated with strong compositional fluctuations and the resulting local structural strain.

We quantified octahedral distortions related to bond angles and octahedral tilting and introduced two structural metrics for the orthorhombic configurations in the Supporting Information (SI, eqs 4 and 5). Octahedral distortion is quantified by the mean-square deviation (MSD) of the X–Sn–X bond angles from the ideal value of 90°, whereas octahedral tilting is quantified by the MSD of the Sn–X–Sn bond angles from the ideal value of 180°. We find that at the intermediate compositions (*x* = 0.5), MSD­(*θ*_X–Sn–X_) exhibits the widest distribution, spanning 1.20–14.53 deg^2^ (Figure S6a), consistent with the maximized number of configurations. The lowest-energy configuration at each composition exhibits an MSD­(*θ*_X–Sn–X_) of only 0.37–4.48 deg^2^, indicating that low-energy configurations are generally associated with a small degree of fluctuation of the X–Sn–X angle. The distribution of Δ*H* in the MSD­(*θ*_X–Sn–X_), MSD­(*θ*_Sn–X–Sn_) space (Figure S6b) shows that high Δ*H* configurations often occur in regions with larger octahedral distortions and larger tilting.

To quantitatively assess the impact of *S*_confi_ on the mixing thermodynamics, three free-energy models *F*_true_, *F*_ideal_, and *F*_min_, as defined in Figure 3a and the Supporting Information, are compared at 300 K. Figure 3b–d shows the composition dependence for the cubic, tetragonal, and orthorhombic phases, and Figures S7–S9 show the corresponding enthalpic and entropic contributions.

**Figure 3:**
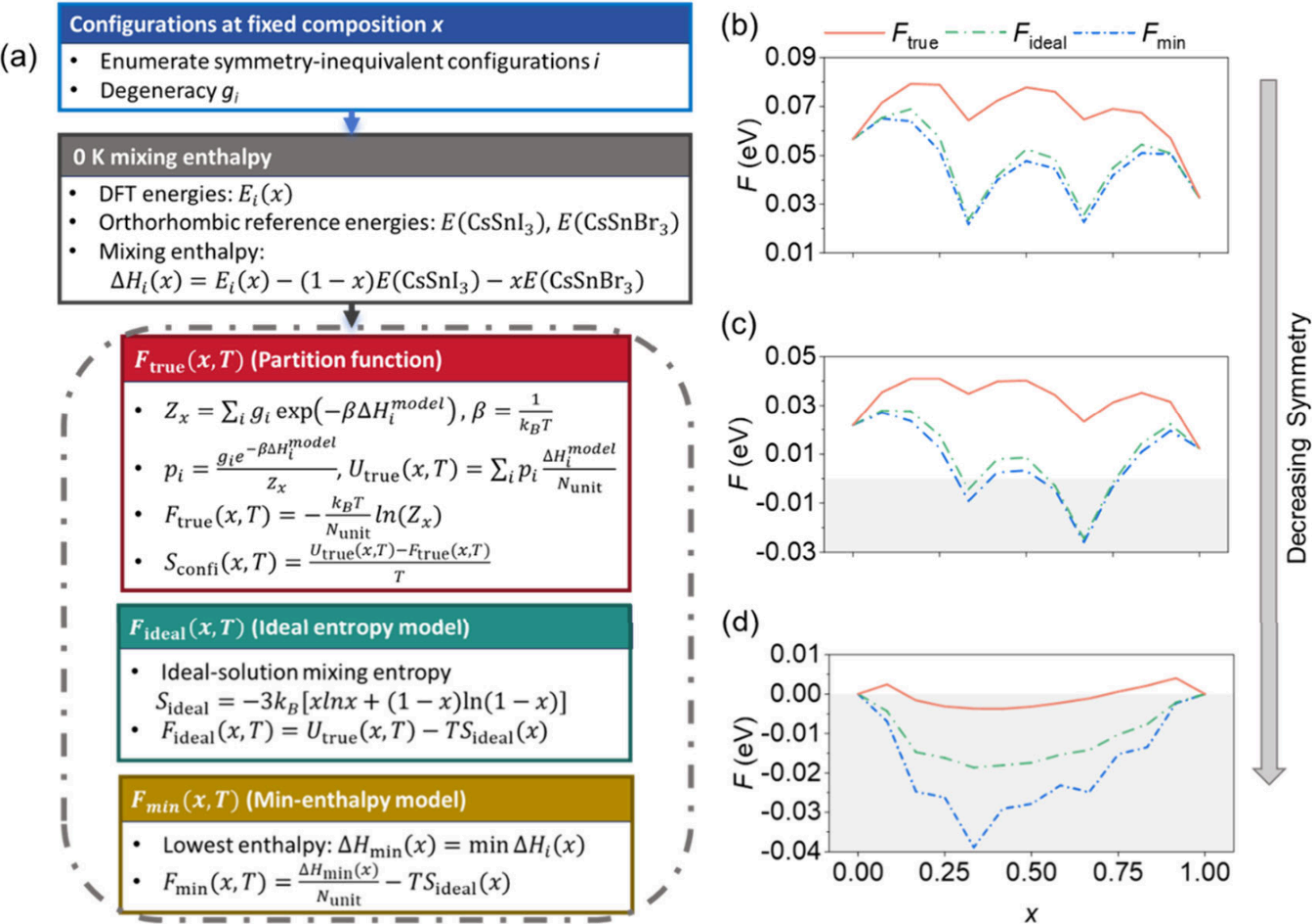
(a) Workflow schematic for configurational averaging and the three free-energy models. Composition dependent free energies of CsSn­(Br_*x*_I_1–*x*_)_3_ at 300 K for the (b) cubic, (c) tetragonal, and (d) orthorhombic phases.

In the cubic and tetragonal phases (Figure 3b,c), *F*_true_ remains positive over the entire composition range, implying that Br/I mixing is thermodynamically unfavorable at 300 K. In contrast, the *F*_true_ curve of the orthorhombic phase (Figure 3d) is noticeably lower. The free energy approaches zero or even becomes slightly negative near intermediate compositions, suggesting that this lowest-symmetry phase becomes thermodynamically miscible at room temperature. The lower curve of free energy in the lower lattice symmetry, such as orthorhombic alloying perovskites, originates from the stronger entropy gain.

By further comparing the three free-energy models, we find that in the cubic phase *F*_true_ is clearly higher than *F*_min_ and *F*_ideal_. This indicates that considering only *U*_min_ or simply adding *S*_ideal_ would substantially underestimate the actual mixing free energy. As the crystal symmetry is decreased, this discrepancy gradually decreases among the three free-energy models. In the orthorhombic phase, the free-energy curves exhibit minimal differences over *x*, reflecting the narrow Δ*H* distribution due to its low-symmetry lattice, which efficiently relaxes local compositional and structural changes. The present free-energy analysis does not include vibrational free energies or anharmonic effects. As shown in Figure S10, we report the composition dependence of the zero-point vibrational energy (ZPE) of the orthorhombic phase, which is evaluated based on the lowest-energy configuration at each composition. Therefore, the curve exhibits local fluctuations rather than a strictly linear trend. However, a fully quantitative treatment of vibrational and anharmonic contributions is beyond the scope of this work.

As shown in Figure 4, the free energies of the three phases at 300 K are compared using three models. For *F*_true_ (Figure 4a), the free energies follow the order orthorhombic < tetragonal < cubic, with the orthorhombic phase having lowest values closest to zero across *x* and cubic phase having highest free energy, indicating unfavorable Br/I mixing despite the consideration of *S*_confi_. When *S*_ideal_ is adopted (Figure 4b), all free-energy curves are further lowered. For the orthorhombic phase, a negative region of *F*_ideal_ appears at intermediate compositions, the tetragonal phase decreases slightly below zero at some compositions, and the cubic phase remains positive overall. It indicates that low-symmetry phases are nearly close to being fully miscible, in contrast to the cubic phase. In the *F*_min_ limit (Figure 4c), the shape of the curves mainly reflects *U*_*min*_. The orthorhombic phase exhibits negative values over the entire *x*, the tetragonal phase is distributed around zero, and the cubic phase remains positive, indicating that the cubic phase is the least favorable phase for alloying.

**Figure 4:**
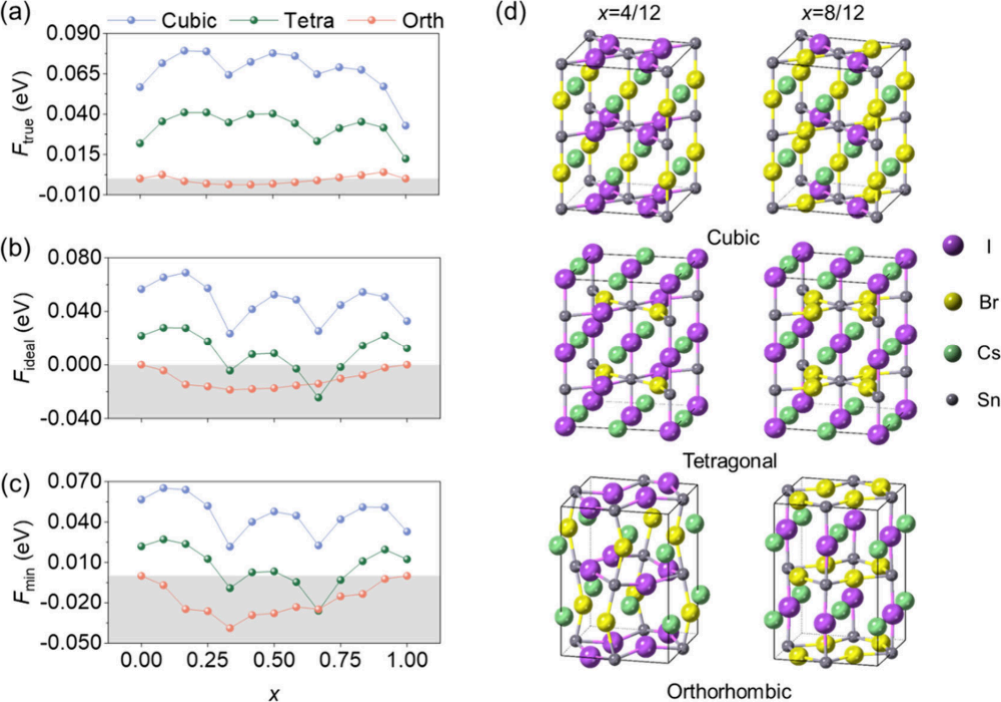
Comparison of the three free-energy models: (a) *F*_true_, (b) *F*_ideal_, and (c) *F*_min_ for CsSn­(Br_*x*_I_1–*x*_)_3_ as a function of *x* obtained at 300 K. (d) Representative lowest-energy configurations corresponding to the local free-energy minima (*x* = 4/12 and 8/12).

In Table S1, we quantify the free-energy differences between phases in units of thermal energy *k*_B_ *T* at 300 K. For Δ*F*_cub‑orth_ = *F*_cub_ – *F*_orth_, the values remain positive throughout the entire composition range, spanning (1.27–3.42)*k*_B_ *T*, which indicates a robust thermodynamic preference for the orthorhombic phase over the cubic phase at 300 K. For Δ*F*_tet‑orth_ = *F*_tet_ – *F*_orth_, the values range from (−0.40 to 1.87)*k*_B_ *T*. Near *x* = 8/12, Δ*F*_tet‑orth_ becomes negative in *F*_ideal_ and *F*_min_ (with *S*_ideal_) whereas it remains positive in *F*_true_ (with *S*_confi_). It suggests that the competition between tetragonal and orthorhombic phases mainly appears near *x* = 8/12, while the orthorhombic phase remains favored. In addition, Figures S11–13 present the lowest-energy configuration at each composition for the three phases. As shown in Figure 4a–c, pronounced local minima appear at *x* = 4/12 and 8/12. The corresponding lowest-energy configurations, illustrated in Figure 4d, exhibit a higher degree of local Br/I ordering. The detailed structural parameters of these configurations are provided in Tables S2–S4.

To assess temperature effects, we evaluated the free energies in the range of 200–400 K using three free-energy models (Figures S14–S16). Increasing the temperature lowers the free energies of all models, indicating enhanced entropic stabilization. The orthorhombic phase exhibits a broader composition range at 400 K where the mixing free energy becomes negative in all three models, suggesting an expanded miscible region at elevated temperatures. For *x* = 0.33–0.67, the orthorhombic phase exhibits negative *F*_true_ at 300–400 K, indicating thermodynamically favorable alloy formation. However, defect formation and Sn^2+^ oxidation may limit the stability and device performance in practical applications.

To assess the difference between simple and thermal averaging, we compared the volumes and *E*_*g*_ values of the three phases. As shown in Figure 5a–c, both the simple averaging volume (*V*_mean_) and the thermal averaging volume (*V*_300 K_) decrease approximately linearly with increasing *x*, following Vegard’s law. At a fixed composition, *V*_300 K_ is slightly smaller than *V*_mean_, particularly in the cubic and tetragonal phases, reflecting the larger Boltzmann weights of lower-volume configurations at 300 K. As a result, thermal averaging leads to a slight shrinkage of the equilibrium volume compared to the simple averaging. Figure S17 shows that Vegard’s law is valid only for the α phase.

**Figure 5:**
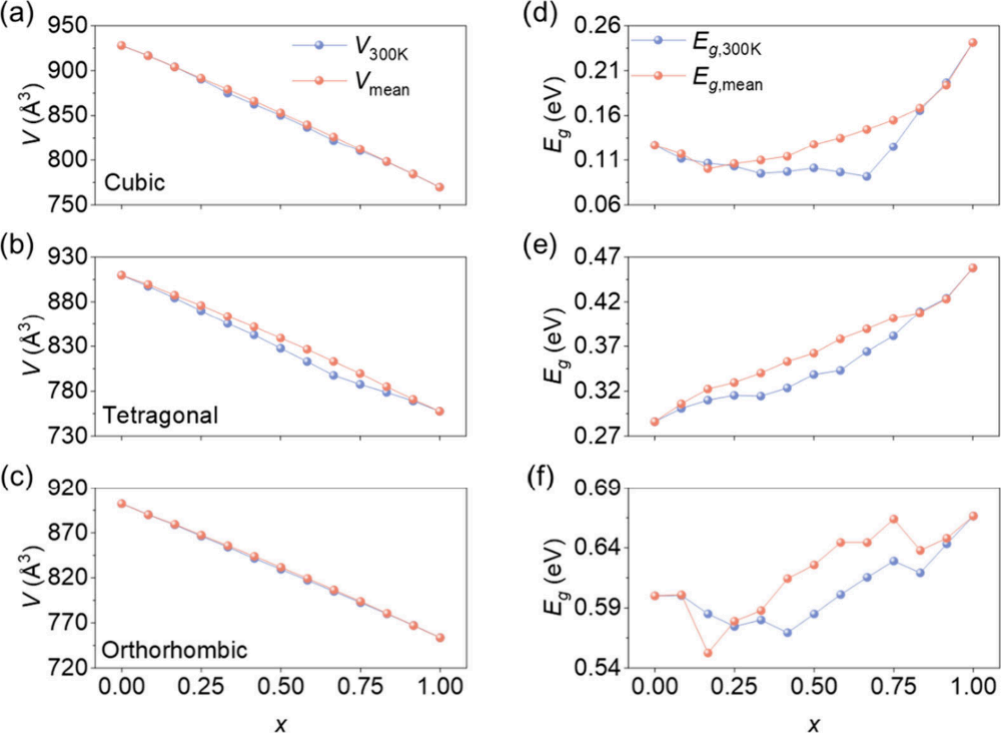
Composition-dependent volume and band gap for CsSn­(Br_*x*_I_1–*x*_)_3_ at 300 K.

Figure 5d–f shows the composition dependence of *E*_*g*_ for the three phases. *E*_*g*_ increases with increasing *x* values in all phases. The simple average band gap (*E*_*g*__,mean_) shows pronounced local fluctuations, reflecting the strong sensitivity of the band edges to local Br/I arrangements. In contrast, the thermal averaging band gap (*E*_*g*__,300 K_) curves are smoother, following clearer band gap bowing because high-energy configurations associated with abnormal band gaps are thermodynamically suppressed. We calculated a projected band structure analysis for the Γ-point valence band maximum (VBM) and conduction band minimum (CBM) for the lowest-energy orthorhombic configuration at each composition and obtained the corresponding Kohn–Sham eigenvalues (Figure S18). The results show that the VBM has contributions from Sn and halides (I/Br) while the CBM is dominated by Sn. With increasing *x*, the halide contribution at the VBM gradually shifts from I to Br. The VBM and CBM eigenvalues show an overall downward shift with increasing *x*. During structural optimization, to preserve the symmetry of the cubic and tetragonal phases, constraints were applied to some atomic coordinates, which restrict octahedral tilting and keep the Sn–X–Sn bond angles closer to 180°. This leads to stronger Sn–X orbital overlap and induces a reduced theoretical band gap. In contrast, the orthorhombic phase was fully relaxed without such constraints and exhibited more pronounced octahedral tilts and weaker orbital overlap, thus showing a larger band gap.

Although *E*_*g*_ still partially reflects finite-size limitations of the 20-atom model, the overall increase in *E*_*g*_ with *x* and the bowing behavior are consistent with previous reports.^36,50^ Islam et al. reported an *E*_*g*_ of 0.44 eV for the cubic phase CsSnI_3_ using DFT calculations^56^ while Sabba et al. measured an optical *E*_*g*_ value of 1.27 eV for the orthorhombic phase CsSnI_3_ and 1.75 eV for the cubic phase CsSnBr_3_.^36^ The calculated *E*_*g*_ values of CsSnI_3_ and CsSnBr_3_ are 0.13 and 0.24 eV in the cubic phase, 0.29 and 0.46 eV in the tetragonal phase, and 0.60 and 0.67 eV in the orthorhombic phase. These results clearly show that *E*_*g*_ (cubic) < *E*_*g*_ (tetragonal) < *E*_*g*_ (orthorhombic) for both end members. The calculated gaps are smaller than the experimental values, particularly for the cubic and tetragonal phases. This discrepancy is mainly due to the underestimation of the *E*_*g*_ by the PBEsol functional and the symmetry constraints applied during structural optimization, which enhance the overlap of the Sn–X orbital.

Park et al. employed the generalized quasi-chemical approximation to evaluate the free-energy competition between the γ and *δ* polymorphs in CsSn­(Br_*x*_I_1–*x*_)_3_ and proposed CsSnI_2_Br as a composition that balances stability and device efficiency.^54^ They also emphasized the importance of geometric factors such as octahedral tilting and Sn–X bond lengths in tuning the electronic properties. In contrast, we construct the partition function by enumerating all nonequivalent configurations, compute the free energy, and connect the phase stability to local distortion metrics. Our results are consistent in that Br substitution modifies geometric parameters and tunes the free-energy trends, providing a thermodynamic basis for composition engineering and property tuning. In practical device applications, external factors, such as pressure, strain, and defects, may further modify phase stability and electronic properties. Previous studies have shown that strain or pressure can tune octahedral tilts and lattice distortions, thereby affecting relative phase energetics and electronic properties,^57,58^ while defects can impact carrier lifetimes and optoelectronic performance.^59–61^

Our results show that the Δ*H* of CsSn­(Br_*x*_I_1–*x*_)_3_ is primarily controlled by local structural distortions and compositional fluctuations whereas global parameters are mainly determined by the Br fraction. Among the three phases, the orthorhombic phase exhibits the lowest mixing free-energy curve and is closest to the thermodynamic miscibility boundary at room temperature. Quantitatively, at 300 K, the orthorhombic phase is thermodynamically preferred over the cubic phase across the entire compositional range, with Δ*F*_cub‑orth_ = (1.27–3.42)*k*_B_ *T* and Δ*F*_tet‑orth_ = (−0.40–1.87)*k*_B_ *T* for the tetragonal phase. This enhanced stability originates from its low-symmetry lattice, which allows more effective local structural relaxation and results in a narrowed energy landscape. Low-energy configurations exhibit markedly smaller octahedral distortion metrics MSD­(θ_X–Sn–X_) of 0.37–4.48 deg^2^ compared with the broader distribution of 0–14.53 deg^2^, and local Br/I compositional fluctuations *σ*(*k*) = 0–0.58 compare with the distribution of 0–2.58. By constructing free-energy models, we demonstrate that simplified approximations based on *U*_min_ or *U*_true_ combined with *S*_ideal_ underestimate the true mixing free energy, particularly for high-symmetry phases with broad energy distributions. These analyses provide useful guidance for the design of MHPs by highlighting low-symmetry strain relaxation, configuration entropy, and the suppression of high-enthalpy configurations through controlled ordering and distortions. These analyses establish links among octahedral distortions, geometric parameters, thermodynamic stability, and band gap tuning.

## Supplementary Material



## Notes

Computational details, geometric optimization procedures, formulas for structural metrics and thermodynamic analysis, supplementary tables (free-energy differences, structural parameters, mixing enthalpies, configurational statistics, and local distortion metrics), and supplementary figures (local structural distortions and geometric descriptors versus *x*, bond-angle statistics, enthalpic and entropic contributions, zero-point vibrational energy, lowest-energy structures, free energies at different temperatures, lattice constants, and composition dependence of Γ-point VBM and CBM eigenvalues and element-projected weights) for CsSn­(Br_*x*_I_1–*x*_)_3_ (PDF)
