# Supplementary material for: Configurational Entropy and Phase Stability in Lead-Free Mixed-Halide CsSn(Br x I1–x )3
Source: J Phys Chem Lett. 2026 Mar 20;17(13):3940–6. doi: 10.1021/acs.jpclett.6c00224 (PMC13051423; doi:10.1021/acs.jpclett.6c00224)
Supplement: Supplementary file 1 [file jz6c00224_si_001.pdf]

Supporting Information for

Configurational Entropy and Phase Stability in Lead-Free Mixed-Halide $CsSn(Br_xI_{1-x})_3$

*Xing Liu, Bowen Wang, Jiacheng Gong, Xuan Chen, Yongqing Cai**

Joint Key Laboratory of the Ministry of Education, Institute of Applied Physics and Materials Engineering, University of Macau, Avenida da Universidade, Taipa, Macau, China;

*Corresponding author. Email: yongqingcai@um.edu.mo

Computational Methods

1. Density functional theory calculations

All calculations were conducted using the Vienna ab initio Simulation Package (VASP)[1,2]. The electron-ion interactions were implemented with the projector augmented-wave (PAW) method, and the exchange-correlation energy was described by PBEsol functional[3,4]. A plane-wave cutoff energy of 450 eV was employed. $CsSn(Br_xI_{1-x})_3$ models were constructed with 20-atom supercells, and a 3×3×2 k-point mesh was adopted for geometric optimization and a 6×6×4 k-point mesh was employed for self-consistent calculation for deriving electronic properties. Phonon calculations were carried out using the Phonopy code with the finite displacement method[5,6].

2. Geometric optimization

For the cubic, tetragonal, and orthorhombic phase structures, we used Spglib to exhaustive search all symmetry-inequivalent $CsSn(Br_xI_{1-x})_3$ configurations in the 20-atom model[7]. The number of configurations is summarized in Table S4. It shows that from the cubic to the orthorhombic phase, the number of data points increases for the given Br content (x) in $CsSn(Br_xI_{1-x})_3$. It reflects the increase in the number of symmetry-inequivalent configurations as the crystal symmetry decreasing.

The geometric optimization for each symmetry-inequivalent configurations was performed with reference to the work of Pan et al[8]. For the cubic phase, only the lattice parameters were allowed to relax while all fractional coordinates were fixed to keep the ideal cubic framework. For the tetragonal phase, similarly, the lattice parameters were relaxed. The fractional coordinates of Cs, Sn and the out-of-plane halide atoms were fixed, while relaxation of the in-plane halide atoms was allowed. Hence, only limited octahedral distortions are introduced, and the tetragonal symmetry is preserved. For the orthorhombic phase, both the lattice parameters and all fractional coordinates were relaxed until the maximum force converged to 0.01 eV/Å. The system could release strain under the lowest symmetry and form the most energetically favorable structures. For both the cubic and tetragonal phases, this constraint leads to residual strain in the system, where some octahedral distortions are suppressed. However, the mixing enthalpy (ΔH) trends remain consistent with those obtained from the fully relaxed orthorhombic phase.

For each configuration, every $[SnX_6]^{4-}$ octahedron is considered as a unit and define k_i as the number of I atoms in the *i*-th octahedron. The standard deviation of the local composition fluctuation is defined as

$$\sigma(k) = \sqrt{\langle (k_i - \langle k \rangle)^2 \rangle} \qquad (1)$$

where $\langle k \rangle$ is denotes the average value of k_i over all octahedra in the configuration.

We collect all Sn-X bond lengths d_j in each configuration and define the average Sn-X bond length as

$$\langle d \rangle = \langle d_j \rangle \qquad (2)$$

and its standard deviation is defined as

$$\sigma(d) = \sqrt{\left\langle \left(d_j - \langle d \rangle\right)^2 \right\rangle} \qquad (3)$$

We also collect all X-Sn-X angles $\theta_{\mathrm{X-Sn-X}}$ and Sn-X-Sn angles $\theta_{\mathrm{Sn-X-Sn}}$ in each configuration and define the mean-square deviation (MSD) as

$$\mathrm{MSD}(\theta_{\mathrm{X-Sn-X}}) = \langle (\theta_{\mathrm{X-Sn-X}} - 90^{\circ})^2 \rangle \qquad (4)$$

$$\mathrm{MSD}(\theta_{\mathrm{Sn-X-Sn}}) = \langle (\theta_{\mathrm{Sn-X-Sn}} - 180^{\circ})^2 \rangle \qquad (5)$$

We further introduce the short-range order (SRO) parameter $Q_{\mathrm{Br|I}}$ and $Q_{I|I}$ to evaluate the local halide short-range ordering behavior and distinguish between homogeneous clustering (Br-I) and heterogeneous clustering (I-I). The eight nearest halogen neighbors for each I atom is counted and the number of surrounding Br (I) atoms is denoted as $n_{\mathrm{Br}}(n_{\mathrm{I}})$. The overall Br/I atomic fraction in the system is defined as

$$c_{\mathrm{Br}} = \frac{N_{\mathrm{Br}}}{N_{\mathrm{X}}} \qquad (6)$$

$$c_{\mathrm{I}} = \frac{N_{\mathrm{I}}}{N_{\mathrm{X}}} \quad (7)$$

where N_{Br} (N_{I}) and N_{X} are the numbers of Br (I) atoms and of all halogen atoms in the model. $Q_{\mathrm{Br|I}}$ and $Q_{\mathrm{I|I}}$ are defined as

$$Q_{\mathrm{Br|I}} = \frac{\frac{n_{\mathrm{Br}}}{8}}{c_{\mathrm{Br}}} \quad (8)$$

$$Q_{\mathrm{I|I}} = \frac{\frac{n_{\mathrm{I}}}{8}}{c_{\mathrm{I}}} \quad (9)$$

For random Br/I mixing, both quantities approach 1. When $\langle Q_{\mathrm{Br|I}} \rangle$ >1, the local Br concentration around I is higher than the global Br concentration, indicating a tendency toward heterogeneous clustering, whereas $\langle Q_{\mathrm{Br|I}} \rangle$ <1 corresponds to a tendency towards homogeneous clustering. Likewise, $\langle Q_{\mathrm{I|I}} \rangle$ >1 indicates a tendency toward I-I aggregation, whereas $\langle Q_{\mathrm{I|I}} \rangle$ <1 indicates suppressed I-I clustering. For each configuration, we take the average value of $\langle Q_{\mathrm{Br|I}} \rangle$ and $\langle Q_{I|I} \rangle$ over all I centered environments, denoted as $\langle Q_{\mathrm{Br|I}} \rangle$ and $\langle Q_{I|I} \rangle$. Definitions of structural metrics used to quantify local distortions are summarized in Table S5.

3. Thermodynamic analysis

3.1 Mixing enthalpy (ΔH)

To evaluate the thermodynamic stability of the $CsSn(Br_xI_{1-x})_3$ alloy, the ΔH is employed to evaluate the possible phase segregation. The ΔH is defined as

$$\Delta H(\mathrm{CsSnBr}_x\mathrm{I}_{1-x}) = E(\mathrm{CsSnBr}_x\mathrm{I}_{1-x}) - (1-x)E(\mathrm{CsSnI}_3) - xE(\mathrm{CsSnBr}_3) \quad (10)$$

where $E(\mathrm{CsSnBr}_x\mathrm{I}_{1-x})$ is the energy of the Br/I mixed $\mathrm{CsSn(Br}_x\mathrm{I}_{1-x})_3$ alloy, and $E(\mathrm{CsSnI}_3)$ and $E(\mathrm{CsSnBr}_3)$ are the energies of the orthorhombic CsSnI_3 and CsSnBr_3, respectively.

3.2 Partition function and free energies

For a given *x*, a set of non-equivalent structures with a degeneracy g_i and a mixing enthalpy ΔH_i^{model} were calculated at 0 K. We use the partition function to describe the thermodynamic behavior of this alloy at a finite temperature *T*. For each *x*, the partition function (Z_x) is defined as

$$Z_x = \sum_i g_i \exp\left(-\beta \Delta H_i^{model}\right) \qquad (11)$$

$$\beta = \frac{1}{k_B T} \qquad (12)$$

where k_B is the Boltzmann constant. The free energy (*F*) at each *x* and temperature *T* is

$$F_\mathrm{true}(x,T) = -\frac{k_B T}{N_\mathrm{unit}} ln(Z_x) \qquad (13)$$

The subscript "true'' indicates that this free-energy model includes all non-equivalent configurations with their degeneracy weights. The 20-atom model contains four formula units, thus N_unit = 4. The Boltzmann occupation probability (p_i) of the *i*-th configuration at temperature *T* and the corresponding thermal average energy (*U*) are

$$p_i = \frac{g_i e^{-\beta \Delta H_i^{model}}}{Z_x} \qquad (14)$$

$$U_\mathrm{true}(x,T) = \sum_i p_i \frac{\Delta H_i^{model}}{N_\mathrm{unit}} \qquad (15)$$

The configurational entropy (S_confi) can be obtained.

$$S_{\text{confi}}(x,T) = \frac{U_{\text{true}}(x,T) - F_{\text{true}}(x,T)}{T} \qquad (16)$$

To access the influence of S_{confi} on the free energy, we also construct two additional free-energy models using the ideal-solution mixing entropy (S_{ideal}).

$$S_{\text{ideal}} = -3k_B[xlnx + (1-x)\ln(1-x)] \qquad (17)$$

In the first model, the energetic part is still used the thermal average energy (U_{true}) obtained from Z_x.

$$F_{\text{ideal}}(x,T) = U_{\text{true}}(x,T) - TS_{\text{ideal}}(x) \qquad (18)$$

In the second model, only the lowest-energy configuration at each composition is retained and the corresponding mixing enthalpy is denoted as $\Delta H_{\text{min}}(x)$. The corresponding free energy is

$$F_{\text{min}}(x,T) = \frac{\Delta H_{\text{min}}(x)}{N_{\text{unit}}} - TS_{\text{ideal}}(x) \qquad (19)$$

Table S1. The free-energy differences between phases for $CsSn(Br_xI_{1-x})_3$ obtained from three free-energy models (F_{true}, F_{ideal} and F_{min}). $\Delta F_{\text{cub-orth}} = F_{\text{cub}} - F_{\text{orth}}$ and $\Delta F_{\text{tet-orth}} = F_{\text{tet}} - F_{\text{orth}}$. All values are reported in units of k_BT at 300K.

Br content (x)	$\Delta F_{\text{cub-orth}}/k_BT$ (F_{true})	$\Delta F_{\text{tet-orth}}/k_BT$ (F_{true})	$\Delta F_{\text{cub-orth}}/k_BT$ (F_{ideal})	$\Delta F_{\text{tet-orth}}/k_BT$ (F_{ideal})	$\Delta F_{\text{cub-orth}}/k_BT$ (F_{min})	$\Delta F_{\text{tet-orth}}/k_BT$ (F_{min})
0	2.19	0.85	2.19	0.85	2.19	0.85
1/12	2.67	1.27	2.69	1.25	2.78	1.31
2/12	3.12	1.65	3.23	1.64	3.42	1.87
3/12	3.17	1.71	2.84	1.31	3.02	1.50

4/12	2.63	1.49	1.63	0.55	2.34	1.15
5/12	2.94	1.68	2.31	1.00	2.67	1.22
6/12	3.13	1.68	2.70	1.00	2.92	1.20
7/12	3.02	1.41	2.48	0.48	2.62	0.72
8/12	2.54	0.94	1.53	-0.40	1.83	-0.04
9/12	2.64	1.18	2.13	0.33	2.21	0.47
10/12	2.52	1.28	2.40	0.85	2.49	0.94
11/12	2.05	1.06	2.05	0.94	2.05	0.84
1	1.27	0.48	1.27	0.48	1.27	0.48

Table S2. Structural parameters and mixing enthalpy for the lowest-energy cubic $CsSn(Br_xI_{1-x})_3$ configurational model.

Br content (x)	Tolerance factor	$\langle d \rangle$ (ang)	$\sigma(d)$ (ang^2)	ΔH (eV)
1/12	1.12	3.06	0.00	0.35
2/12	1.12	3.05	0.00	0.40
3/12	1.12	3.03	0.00	0.38
4/12	1.11	3.01	0.01	0.28
5/12	1.11	3.00	0.01	0.37
6/12	1.12	2.98	0.01	0.41
7/12	1.12	2.97	0.01	0.39
8/12	1.12	2.95	0.01	0.29
9/12	1.12	2.94	0.01	0.34
10/12	1.13	2.92	0.00	0.34
11/12	1.12	2.90	0.00	0.29

Table S3. Structural parameters and mixing enthalpy for the lowest-energy tetragonal $CsSn(Br_xI_{1-x})_3$ configurational model.

Br content (x)	Tolerance factor	$\langle d \rangle$ (ang)	$\sigma(d)$ (ang^2)	ΔH (eV)
1/12	1.11	3.09	0.00	0.20
2/12	1.12	3.08	0.00	0.23
3/12	1.12	3.06	0.01	0.22
4/12	1.12	3.04	0.01	0.16
5/12	1.12	3.03	0.01	0.22
6/12	1.12	3.02	0.01	0.23
7/12	1.12	3.00	0.01	0.19
8/12	1.12	2.98	0.01	0.09
9/12	1.12	2.97	0.01	0.16
10/12	1.12	2.95	0.00	0.18
11/12	1.12	2.93	0.00	0.17

Table S4. Structural parameters and mixing enthalpy for the lowest-energy orthorhombic $CsSn(Br_xI_{1-x})_3$ configurational model.

Br content (x)	Tolerance factor	$\langle d \rangle$ (ang)	$\sigma(d)$ (ang)	$\mathrm{MSD}(\theta_{\mathrm{X-Sn-X}})$ (deg^2)	$\mathrm{MSD}(\theta_{\mathrm{Sn-X-Sn}})$ (deg^2)	ΔH (eV)
1/12	1.07	3.11	0.00	1.72	632.55	0.06
2/12	1.07	3.09	0.01	1.82	629.67	0.04
3/12	1.06	3.08	0.01	2.37	618.14	0.07
4/12	1.06	3.06	0.01	1.47	604.71	0.04
5/12	1.06	3.05	0.01	2.85	610.24	0.09
6/12	1.08	3.02	0.01	2.57	511.66	0.10
7/12	1.07	3.01	0.01	4.48	556.65	0.12
8/12	1.12	2.98	0.01	0.37	550.45	0.10
9/12	1.09	2.97	0.01	3.82	510.04	0.11
10/12	1.11	2.95	0.01	0.76	468.92	0.09
11/12	1.08	2.94	0.00	1.46	492.57	0.08

Table S5. Total number of Br/I configurations and symmetry-inequivalent configurations for $CsSn(Br_xI_{1-x})_3$ in the 20-atom model for each phase. All symmetry-inequivalent configurations were structurally optimized.

Br content (x)	Total configurations	Symmetry-inequivalent configurations (cubic)	Symmetry-inequivalent configurations (tetragonal)	Symmetry-inequivalent configurations (orthorhombic)
0	1	1	1	1
1/12	12	2	2	2
2/12	66	9	9	14
3/12	220	14	14	30
4/12	495	32	34	77
5/12	792	36	40	104
6/12	924	50	56	136
7/12	792	36	40	104
8/12	495	32	34	77
9/12	220	14	14	30
10/12	66	9	9	14
11/12	12	2	2	2
1	1	1	1	1
Sum	4096	238	256	592

Table S6. Definitions of structural metrics used to quantify local distortions in $CsSn(Br_xI_{1-x})_3$.

Metrics	Definitions
$\sigma(k)$	$\sigma(k) = \sqrt{\langle (k_i - \langle k \rangle)^2 \rangle}$
$\langle d \rangle$	$\langle d \rangle = \langle d_j \rangle$
$\sigma(d)$	$\sigma(d) = \sqrt{\left\langle \left(d_j - \langle d \rangle \right)^2 \right\rangle}$
$\mathrm{MSD}(\theta_{\mathrm{X-Sn-X}})$	$\mathrm{MSD}(\theta_{\mathrm{X-Sn-X}}) = \langle (\theta_{\mathrm{X-Sn-X}} - 90°)^2 \rangle$
$\mathrm{MSD}(\theta_{\mathrm{Sn-X-Sn}})$	$\mathrm{MSD}(\theta_{\mathrm{Sn-X-Sn}}) = \langle (\theta_{\mathrm{Sn-X-Sn}} - 180°)^2 \rangle$
$Q_{\mathrm{Br\|I}}$	$c_{\mathrm{Br}} = \frac{N_{\mathrm{Br}}}{N_{\mathrm{X}}}$ $Q_{\mathrm{Br\|I}} = \frac{\frac{n_{\mathrm{Br}}}{8}}{c_{\mathrm{Br}}}$
$Q_{\mathrm{I\|I}}$	$c_{\mathrm{I}} = \frac{N_{\mathrm{I}}}{N_{\mathrm{X}}}$ $Q_{\mathrm{I\|I}} = \frac{\frac{n_{\mathrm{I}}}{8}}{c_{\mathrm{I}}}$

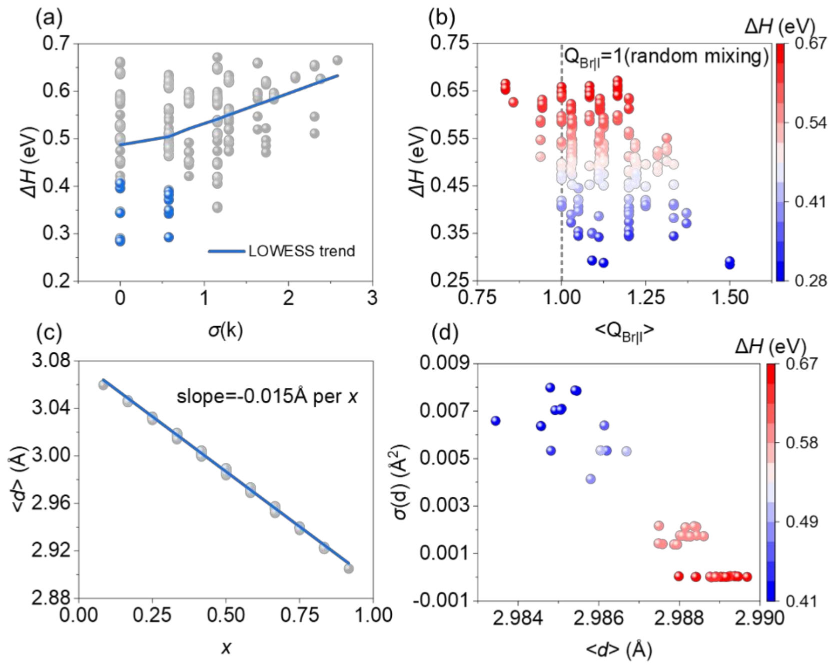

Figure S1. Correlations between local composition, SRO, global geometry parameters, and ΔH for cubic $CsSn(Br_xI_{1-x})_3$. (a) ΔH versus $\sigma(k)$; grey and blue spheres denote all non-equivalent configurations and the lowest-energy configurations at each composition, respectively. The blue line shows a LOWESS smoothing (fraction=0.6). (b) $\langle Q_{\mathrm{Br|I}}\rangle$ versus ΔH. (c) $\langle d\rangle$ versus x; grey spheres denote all non-equivalent configurations, while the blue line indicates a linear fit to $\langle d\rangle$. (d) Distribution of ΔH for $Cs_4Sn_4I_6Br_6$ (x=6/12) configurations in the $(\langle d\rangle,\sigma(\mathrm{d}))$ space.

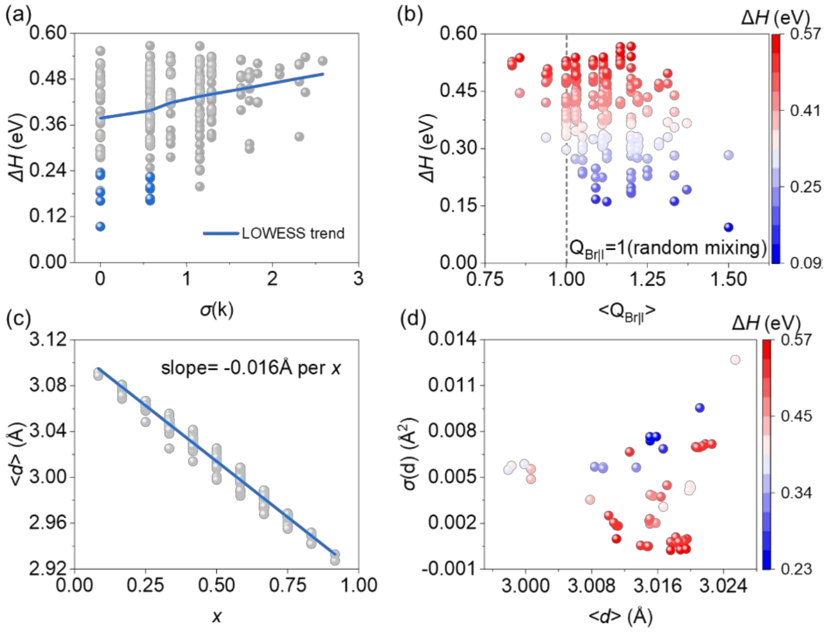

Figure S2. Correlations between local composition, SRO, global geometry parameters, and ΔH for tetragonal $CsSn(Br_xI_{1-x})_3$. (a) ΔH versus $\sigma(k)$; grey and blue spheres denote all non-equivalent configurations and the lowest-energy configurations at each composition, respectively. The blue line shows a LOWESS smoothing (fraction=0.6). (b) $\langle Q_{\mathrm{Br|I}} \rangle$ versus ΔH. (c) $\langle d \rangle$ versus *x*; grey spheres denote all non-equivalent configurations, while the blue line indicates a linear fit to $\langle d \rangle$. (d) Distribution of ΔH for $Cs_4Sn_4I_6Br_6$ (*x*=6/12) configurations in the $(\langle d \rangle, \sigma(\mathrm{d}))$ space.

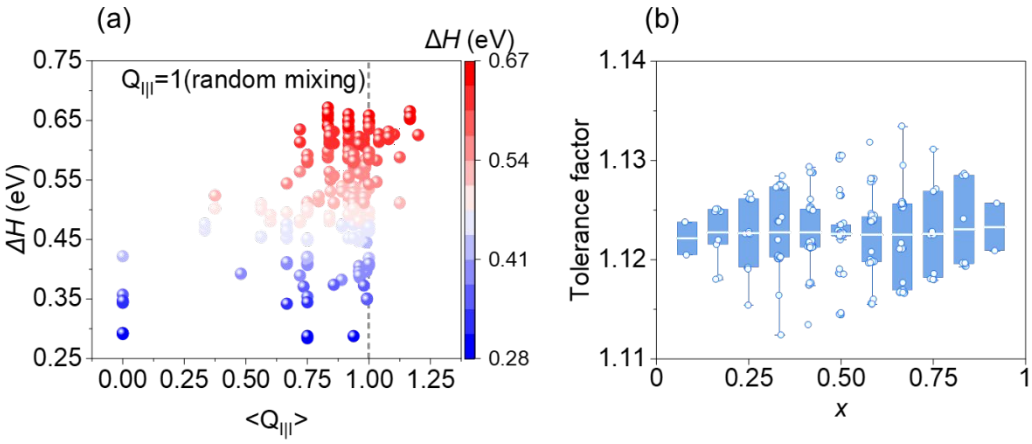

Figure S3. (a) Correlation between ΔH and SRO parameter $\langle Q_{\mathrm{I|I}} \rangle$ in the cubic $CsSn(Br_xI_{1-x})_3$ phase. (b) Geometric tolerance factor versus x.

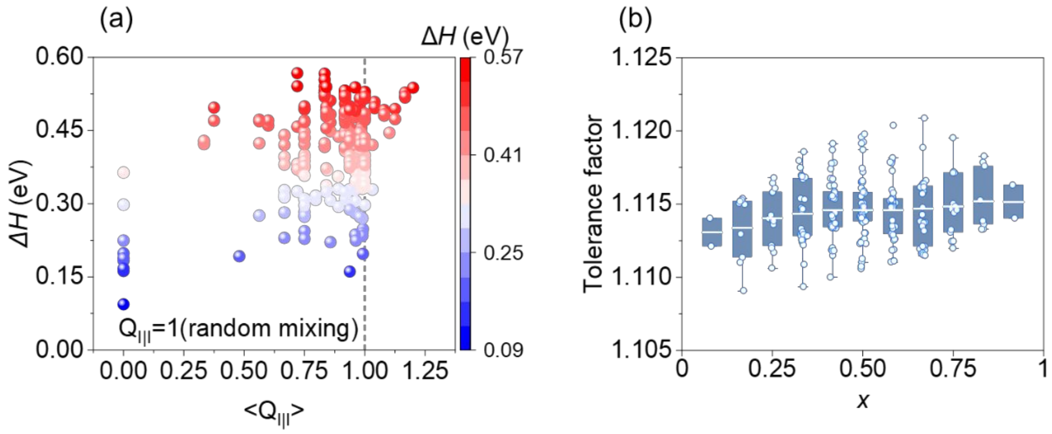

Figure S4. (a) Correlation between ΔH and SRO parameter $\langle Q_{\mathrm{I|I}} \rangle$ in the tetragonal $CsSn(Br_xI_{1-x})_3$ phase. (b) Geometric tolerance factor versus x.

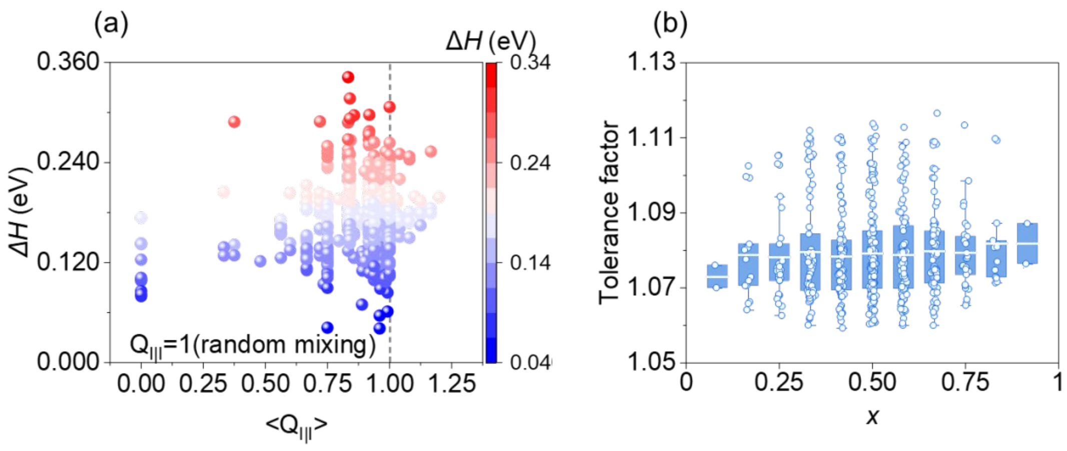

Figure S5. (a) Correlation between ΔH and SRO parameter $\langle Q_{\mathrm{I|I}} \rangle$ in the orthorhombic $CsSn(Br_xI_{1-x})_3$ phase. (b) Geometric tolerance factor versus x.

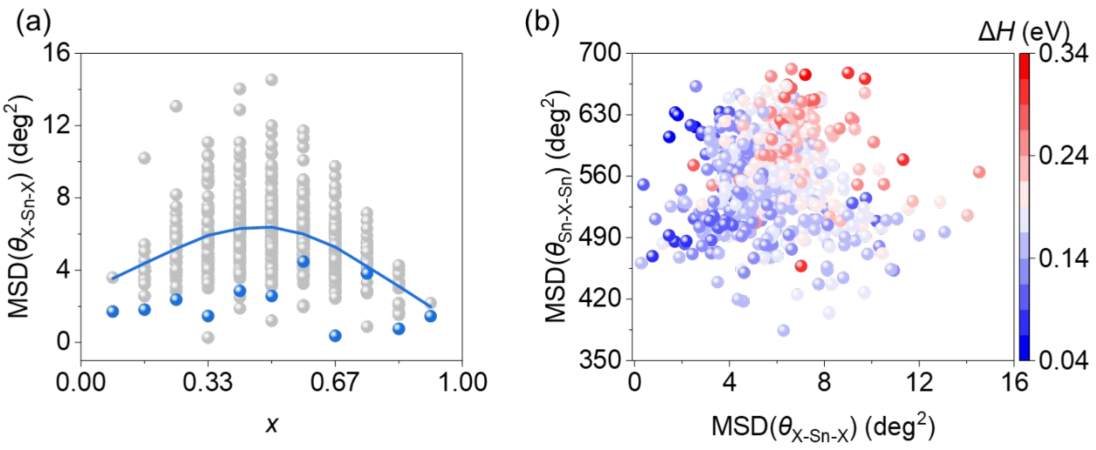

Figure S6. Correlations between Br/I composition, octahedral distortion, octahedral tilting, and ΔH for orthorhombic $CsSn(Br_xI_{1-x})_3$. (a) $\mathrm{MSD}(\theta_{\mathrm{X-Sn-X}})$ versus x; grey and blue spheres denote all non-equivalent configurations and the lowest-energy configurations at each composition, respectively. The blue line shows a LOWESS smoothing (fraction=0.6). (b) Distribution of ΔH orthorhombic configurations in the $(\mathrm{MSD}(\theta_{\mathrm{X-Sn-X}}),\mathrm{MSD}(\theta_{\mathrm{Sn-X-Sn}}))$ space.

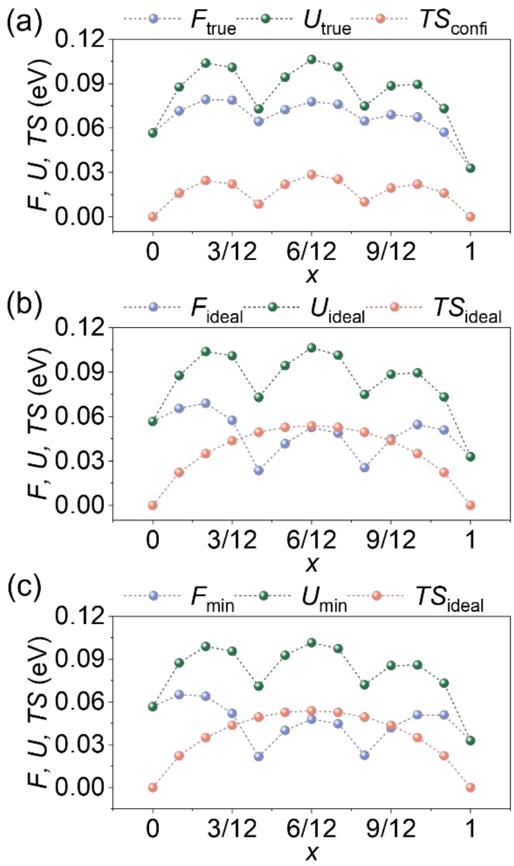

Figure S7. Enthalpic and entropic contributions used in the three free-energy models for the cubic $CsSn(Br_xI_{1-x})_3$ phase at 300 K.

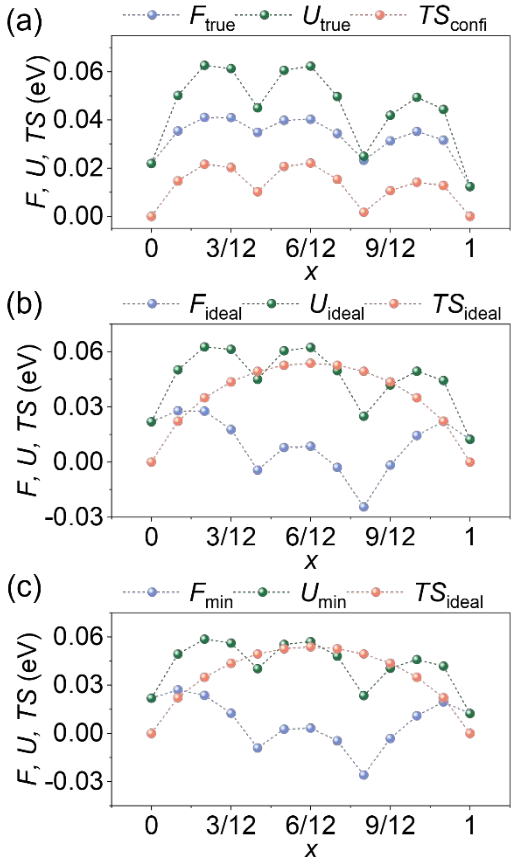

Figure S8. Enthalpic and entropic contributions used in the three free-energy models for the tetragonal $CsSn(Br_xI_{1-x})_3$ phase at 300 K.

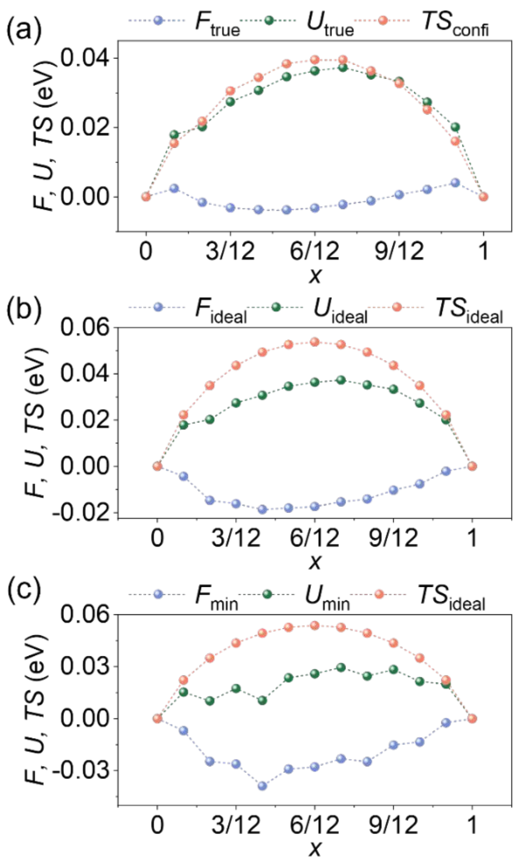

Figure S9. Enthalpic and entropic contributions used in the three free-energy models for the orthorhombic $CsSn(Br_xI_{1-x})_3$ phase at 300 K.

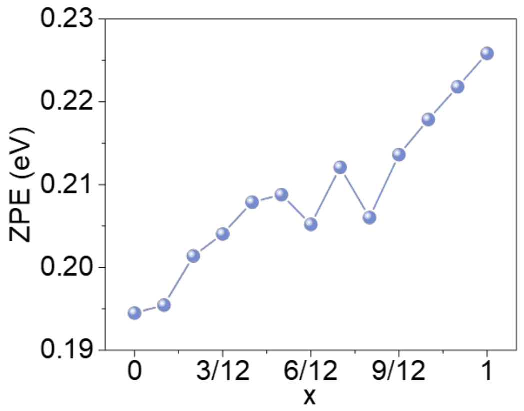

Figure S10. Zero-point vibrational energy (ZPE) of orthorhombic $CsSn(Br_xI_{1-x})_3$ as a function of *x*.

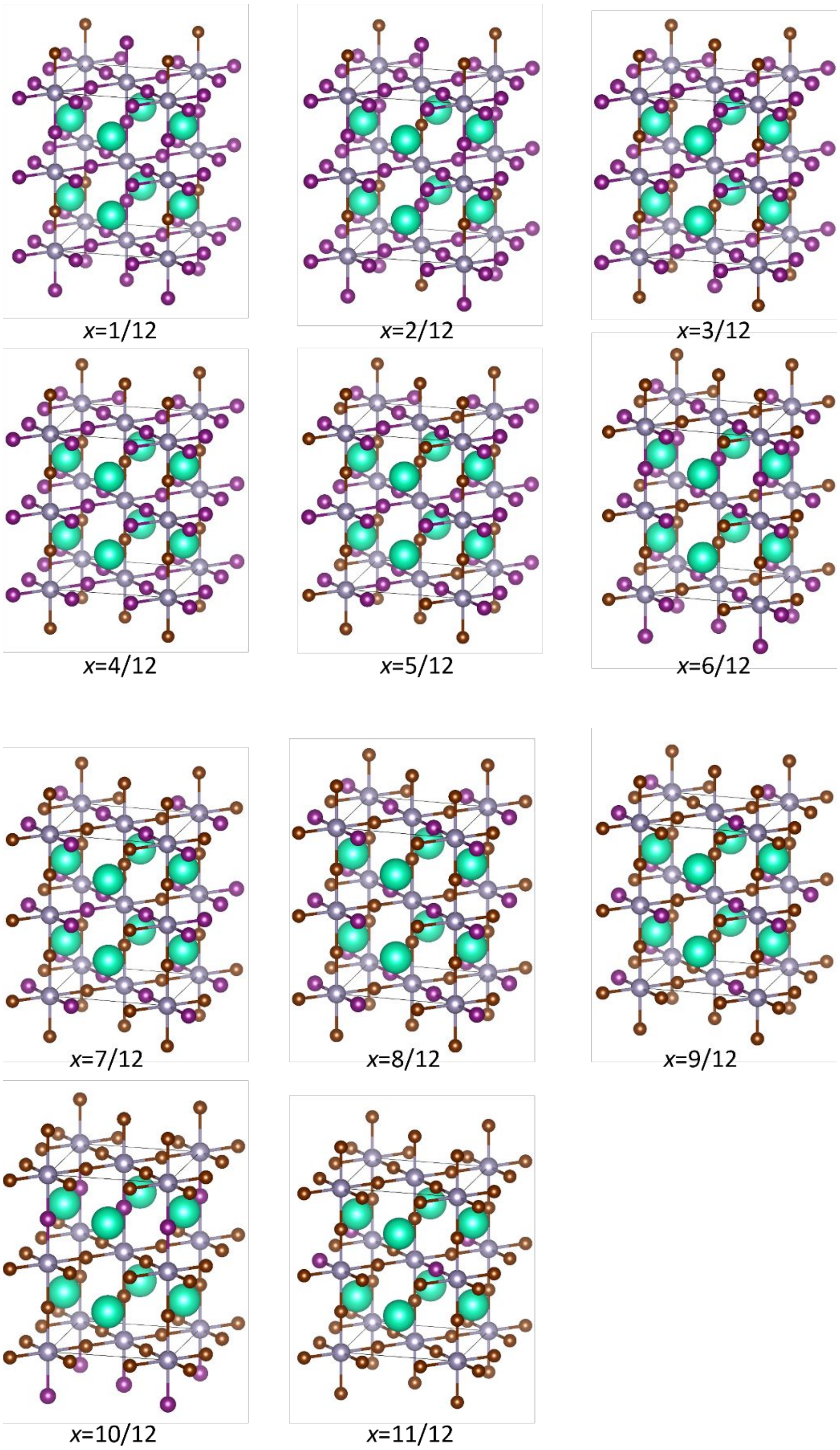

Figure S11. Lowest-energy cubic configurations of $CsSn(Br_xI_{1-x})_3$.

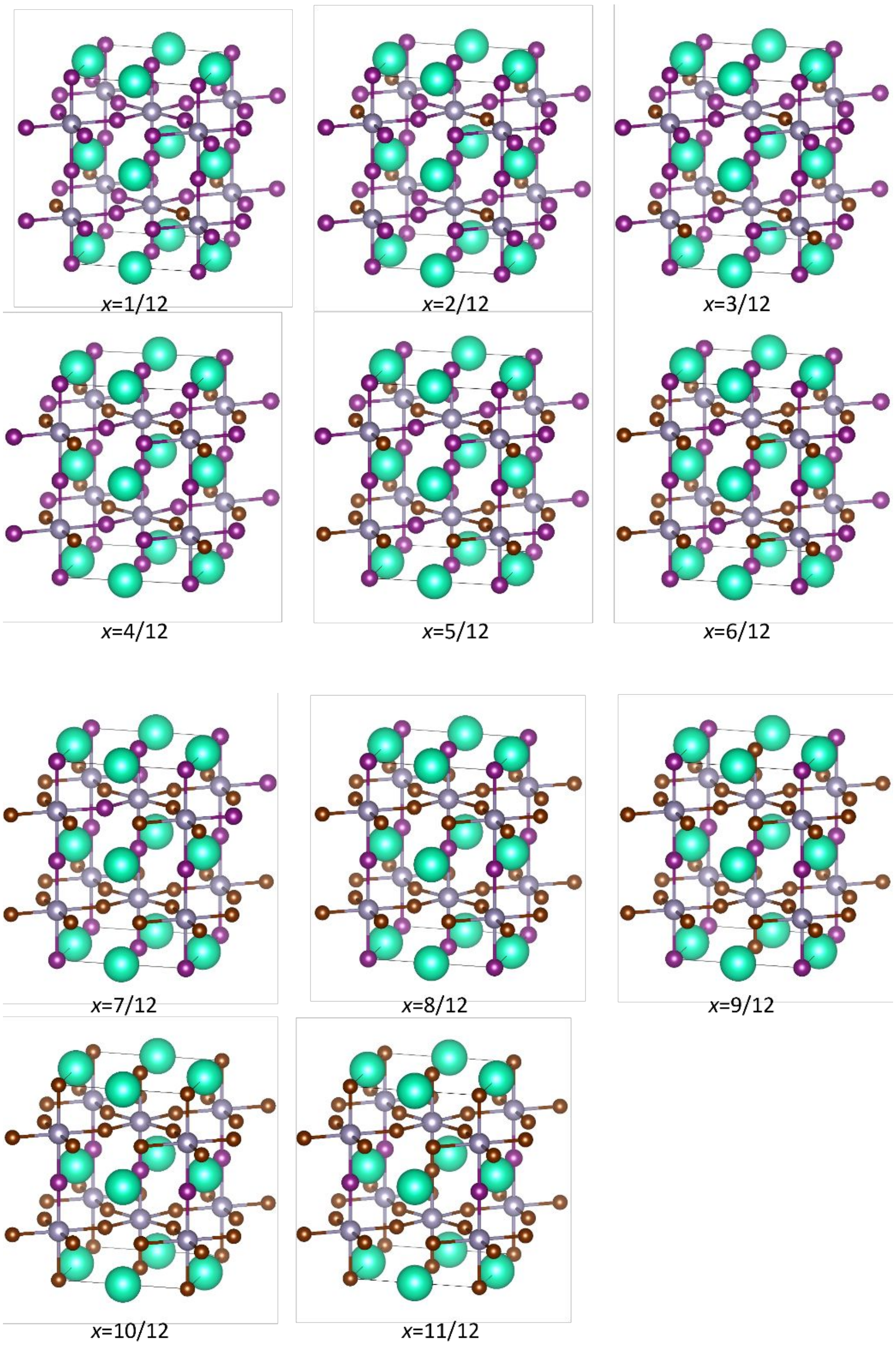

Figure S12. Lowest-energy tetragonal configurations of $CsSn(Br_xI_{1-x})_3$.

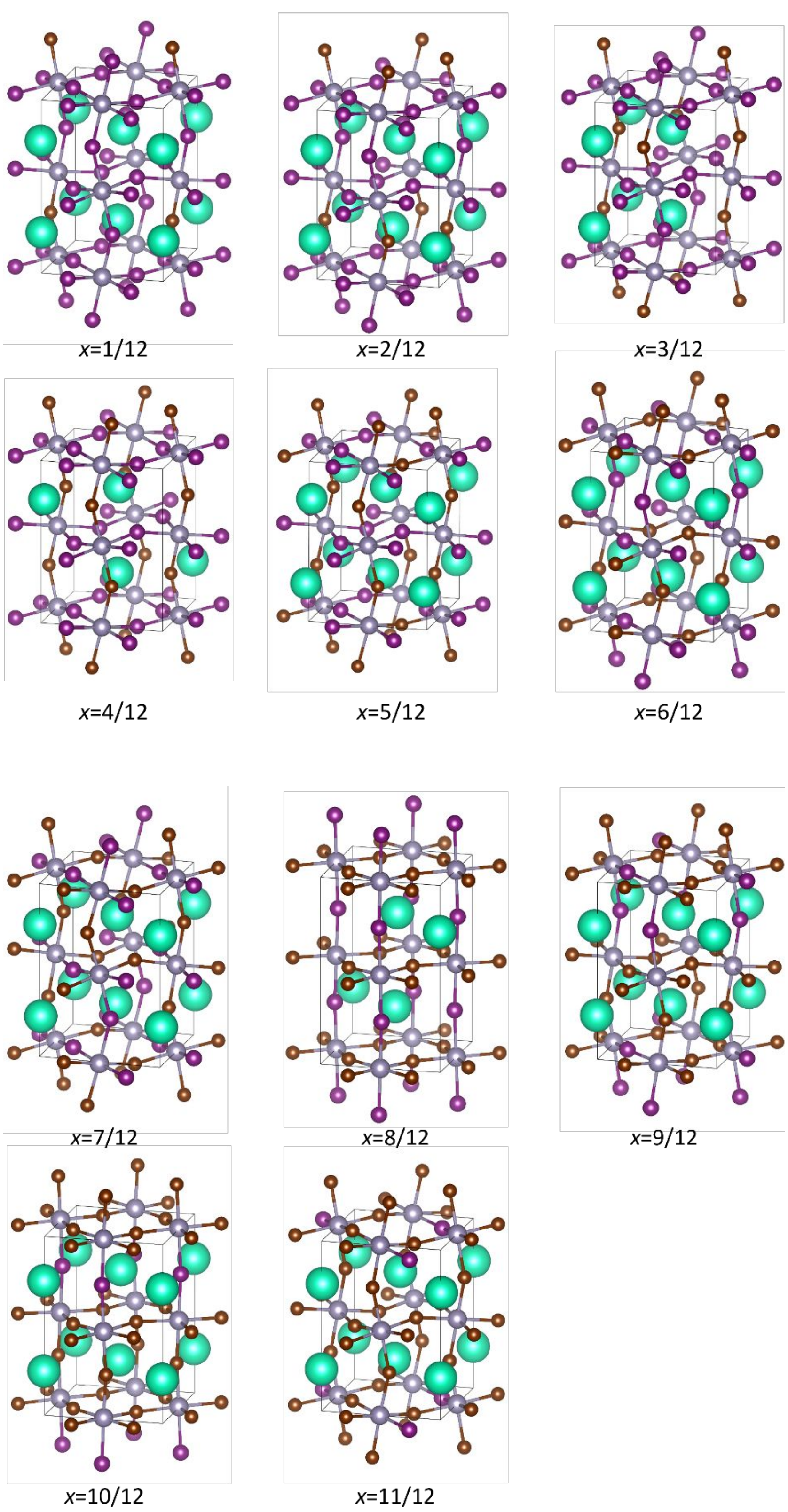

Figure S13. Lowest-energy orthorhombic configurations of $CsSn(Br_xI_{1-x})_3$.

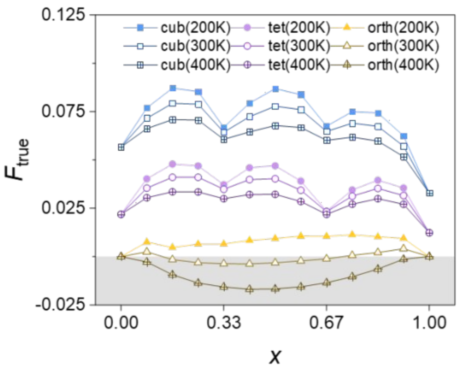

Figure S14. Comparison of F_{true} for cubic, tetragonal and orthorhombic $CsSn(Br_xI_{1-x})_3$ as a function of x at 200-400 K.

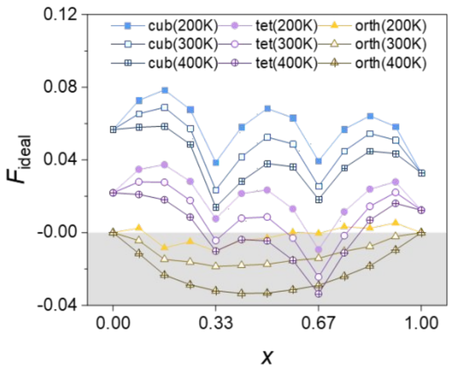

Figure S15. Comparison of F_{ideal} for cubic, tetragonal and orthorhombic $CsSn(Br_xI_{1-x})_3$ as a function of x at 200-400 K.

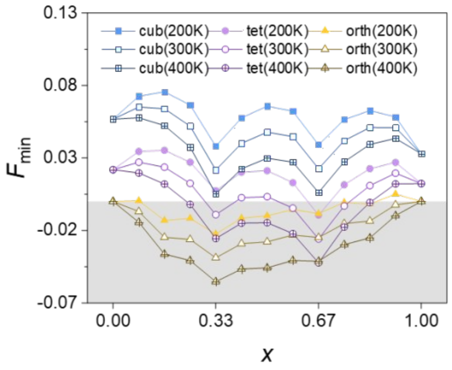

Figure S16. Comparison of F_{min} for cubic, tetragonal and orthorhombic $CsSn(Br_xI_{1-x})_3$ as a function of x at 200-400 K.

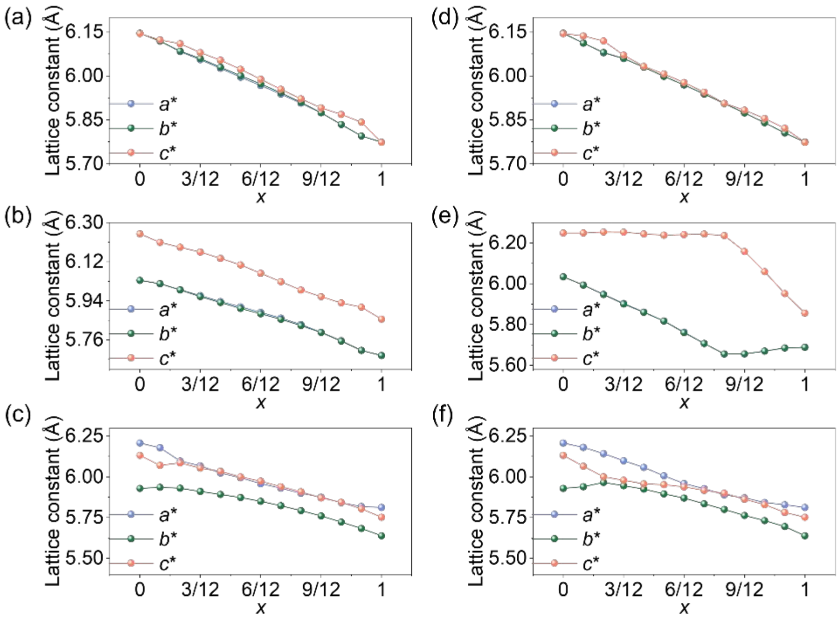

Figure S17. Composition dependence of the lattice constant of $CsSn(Br_xI_{1-x})_3$. (a-c) simple averaging and (d-f) thermal averaging values at 300 K.

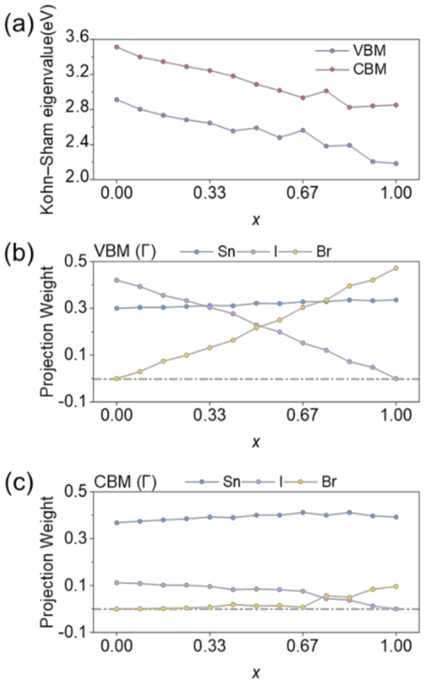

Figure S18. Composition dependence of Γ-point VBM and CBM (a) Kohn–Sham eigenvalues and (b) element-projected weights of VBM and (c) CBM for the lowest-energy orthorhombic configuration at each composition.

REFERENCES

(1) Kresse, G.; Furthmüller, J. Efficient Iterative Schemes for Ab Initio Total-Energy Calculations Using a Plane-Wave Basis Set. *Phys. Rev. B* **1996**, *54* (16), 11169–11186. https://doi.org/10.1103/PhysRevB.54.11169.

(2) Kresse, G.; Joubert, D. From Ultrasoft Pseudopotentials to the Projector Augmented-Wave Method. *Phys. Rev. B* **1999**, *59* (3), 1758–1775. https://doi.org/10.1103/PhysRevB.59.1758.

(3) Blöchl, P. E. Projector Augmented-Wave Method. *Phys. Rev. B* **1994**, *50* (24), 17953–17979. https://doi.org/10.1103/PhysRevB.50.17953.

(4) Perdew, J. P.; Ruzsinszky, A.; Csonka, G. I.; Vydrov, O. A.; Scuseria, G. E.; Constantin, L. A.; Zhou, X.; Burke, K. Restoring the Density-Gradient Expansion for Exchange in Solids and Surfaces. *Phys. Rev. Lett.* **2008**, *100* (13), 136406. https://doi.org/10.1103/PhysRevLett.100.136406.

(5) Togo, A. First-Principles Phonon Calculations with Phonopy and Phono3py. *J. Phys. Soc. Jpn.* **2023**, *92* (1), 012001. https://doi.org/10.7566/JPSJ.92.012001.

(6) Togo, A.; Chaput, L.; Tadano, T.; Tanaka, I. Implementation Strategies in Phonopy and Phono3py. *J. Phys.: Condens. Matter* **2023**, *35* (35), 353001. https://doi.org/10.1088/1361-648X/acd831.

(7) Togo, A.; Shinohara, K.; Tanaka, I. Spglib: A Software Library for Crystal Symmetry Search. *Sci. Technol. Adv. Mater.: Methods* **2024**, *4* (1), 2384822. https://doi.org/10.1080/27660400.2024.2384822.

(8) Pan, F.; Zhai, J.; Chen, J.; Yang, L.; Dong, H.; Yuan, F.; Jiang, Z.; Ren, W.; Ye, Z.-G.; Zhang, G.-X.; Li, J. Mixed-Halide Perovskite Alloys $CsPb(I_{1-x}Br_x)_3$ and $CsPb(Br_{1-x}Cl_x)_3$: New Insight of Configurational Entropy Effect from First-Principles and Phase Diagrams. *Chem. Mater.* **2024**, *36* (8), 3957–3969. https://doi.org/10.1021/acs.chemmater.4c00571.
